# Occlusion of the Os Tincae in a Case of Labor, and Successful Delivery by Incision

**Published:** 1851-10

**Authors:** W. H. Reynale

**Affiliations:** Dansville


ART. III. — *Occlusion of the os tincae in a case of Labor, and successful delivery by incision;* also a case of removal of sixty-five tumors in one operation. Reported, in a letter to Prof. Hamilton, by W. H. REYNALE, M. D., of Dansville.

Three weeks ago I was sent for to visit a lady, Mrs. Goodrich, of this place, in labor, with her first child, of twenty-four hours duration. When I arrived the pains were hard, frequent, and pressing down. Upon examination, I could find no os uteri. I waited four hours in hopes time might develope one, and made several minute examinations during that period of time, but each examination satisfied me there was no os nor cervix uteri; every thing was perfectly smooth where the natural opening ought to have been, and I could feel the child's head distinctly pressing down upon the soft parts. I now told the husband and ladies present, the situation of the patient, and requested another physician to be called in. Dr. Hovey was sent for; and subsequently Dr. Cook saw her — *all agreed* that the os and cervix uteri were wanting; and that there was no natural opening for the child to pass through. Before Dr. Cook saw the patient, Dr. Hovey and myself made use of two vaginal speculums, one of gutta percha, and the other of German silver, both excellent instruments; we saw distinctly every part of the vagina, examined minutely the back, sides, and upper part, and where the os uteri naturally ought to have been, here we examined with a probe introduced through the speculum, but nothing in the shape of the smallest opening could be found. Of course nothing could be done but to cut and make an artificial opening; this was done nine hours after I first saw the patient. I wound a spear-pointed bistoury within half an inch of its point, and by carrying it between my index and my middle fingers, I made an incision of about two inches in length at the exact spot where I supposed the os uteri naturally ought to have been: water followed the incision. The opening dilated upon the contraction of the womb. The incision continued to dilate very much as the natural os would upon the contraction of the womb, and at the expiration of two and a half hours she was safely delivered of a healthy female child weighing nine and a half pounds. The patient has recovered without one bad symptom.

Fourteen months ago, I was desired to see a son of Mr. — Murrays, ten years of age, of Ossian, Allegany county, eight miles from this place. I found the boy small of stature, very pale, with a large tumor on the left side of the neck. He looked like a child with two heads, only the tumor was the largest. It occupied the whole space from the root of the ear to the acromion process of the scapula; filled up every thing from the spinous processes of the cervical vertebra to the clavicle, run from the ear along the lower side of the cheek and jaw bone, to beyond the trachea on the opposite side of the neck; so that in fact it filled up and occupied a little more than the entire space on the left side of the neck, and threw the head on the opposite shoulder. I removed the entire mass, with the assistance of Doctors Endress and Patchin; we took out sixty-five distinct tumors, attached together by cellular substance from the size of a goose egg, and larger, down to that of a marowfat pea; they were of a fatty substance, I think. They filled a half gallon jar after their removal. The patient recovered so as to enjoy tolerably good health for eight months, when a number of small tubercles began to form around the margin of the old extirpated tumor, most were on the shoulder, some near the ear, and a few near the clavicle and spine; all were on the circumference, and none on the cicatrix. He soon after this began to complain of a pain in his left side, just below and beneath the false ribs. His father fetched him to my house; upon an examination I found a tumor of a large size under and coming out from beneath the ribs, and quite painful to the touch. I told the father I could do nothing for his son, the disease was of a malignant nature, and that he would die. He lived twelve months from the time of the operation, and died. His father sent me word at the time of his death, according to agreement; but in consequence of my being from home at the time, I did not get the information, and no post mortem examination was made, which I much regretted.

We kept our little patient fully under the influence of chloroform during the whole of the operation, which lasted nearly two hours, but full one half of the time we desisted from cutting, and applied restoratives, as the patient sunk low. You may probably think we used the chloroform too long; but we weighed the chances of the patient in our own minds, for and against the influence of chloroform on his nervous system, before the operation. We believed he would sink under the influence of the operation without the use of the chloroform, and that he could but die with its use. We decided, after mature consultation, to use the chloroform — it done upon the whole, well — the patient had no knowledge of pain.

After the *entire mass, externally, was removed*, we discovered that a tumor of the size of a black walnut, was lying under the clavicle; this we removed without much difficulty; then another was discovered lower down, of about the same size; this I seized with two tenaculums, one in each extremity of the tumor, an assistant held them firmly and made a little pressure upwards, when, partly by dissecting, and partly with the handle of the scalpel, and partly with my fingers, I succeeded in extracting this also; but still there was another one of the same magnitude, lying yet deeper and beneath the last one — this laid below the second rib; it was also seized with two tenaculums, and firmly held by an assistant, until partly with the handle of the knife, but mostly with my fingers, and a very little dissection with the knife when it could not possibly be avoided, I succeeded in getting *it all out* — no more could be felt. It is unnecessary to describe the situation of the patient, from day to day, during his convalescence; for the first few days he vacillated between hope and fear, but the powers of nature came to our assistance, and the patient recovered so far as to enjoy tolerably good health for eight months; ate, and slept well, the countenance improved, and he looked better than he had for two years before the operation. The head resumed its natural position, and the rotary motion was good. The patient went to school some two or three month during this time. This enormous mass was only two years in growing; when it was first discovered it was of the size of a filbert, or smaller, and situated directly at the root of the ear. Mr. Murray says many applications were used to discuss the tumor, but they all (he thinks) hastened its growth.

